# The Electronic McPhail Trap

**DOI:** 10.3390/s141222285

**Published:** 2014-11-25

**Authors:** Ilyas Potamitis, Iraklis Rigakis, Konstantinos Fysarakis

**Affiliations:** 1 Department of Music Technology & Acoustics, Technological Educational Institute of Crete, E. Daskalaki Perivolia 74100, Rethymno Crete, Greece; 2 Department of Electronics, Technological Educational Institute of Crete, Romanou 3-Chalepa, Chania 73133, Greece; E-Mail: rigakis@chania.teicrete.gr; 3 Department of Electrical and Computer Engineering, Technical University of Crete, Kounoupidiana, Chania 73100, Greece; E-Mail: kfysarakis@isc.tuc.gr

**Keywords:** McPhail trap, automatic insect trap, precision agriculture

## Abstract

Certain insects affect cultivations in a detrimental way. A notable case is the olive fruit ﬂy (*Bactrocera oleae* (*Rossi*)), that in Europe alone causes billions of euros in crop-loss/per year. Pests can be controlled with aerial and ground bait pesticide sprays, the efficiency of which depends on knowing the time and location of insect infestations as early as possible. The inspection of traps is currently carried out manually. Automatic monitoring traps can enhance efficient monitoring of flying pests by identifying and counting targeted pests as they enter the trap. This work deals with the hardware setup of an insect trap with an embedded optoelectronic sensor that automatically records insects as they fly in the trap. The sensor responsible for detecting the insect is an array of phototransistors receiving light from an infrared LED. The wing-beat recording is based on the interruption of the emitted light due to the partial occlusion from insect's wings as they fly in the trap. We show that the recordings are of high quality paving the way for automatic recognition and transmission of insect detections from the field to a smartphone. This work emphasizes the hardware implementation of the sensor and the detection/counting module giving all necessary implementation details needed to construct it.

## 1. Introduction

**Figure 1.**
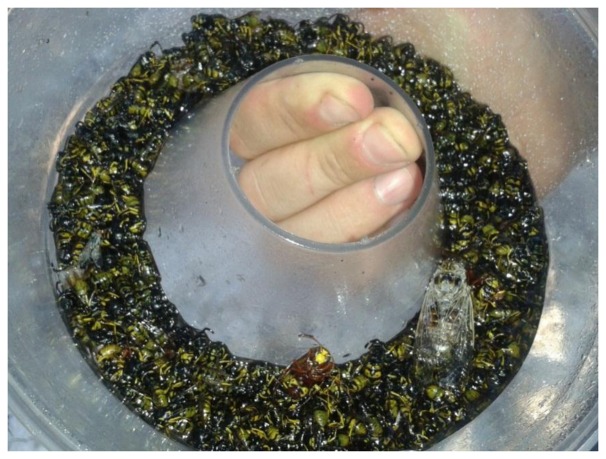
Insects in a standard McPhail trap. Identifying and counting the olive flies can be difficult.

Olive cultivation is widespread throughout the Mediterranean countries and is vital not only for the rural economy but also for the world market. Approximately 2.5 million producers (one third of EU farmers) are involved. The EU is the leading world olive producer, accounting for 80% (>2 million tonnes) [1]. However this sector is continuously under the threat of an extremely destructive insect pest: *Bactrocera oleae* (*Diptera: Tephritidae*), the olive fruit fly. Each year it accounts for more than 30% destruction of all Mediterranean olive crops, *i.e.* losses of almost €3.0 billion [2,3]. In Europe the olive fruit fly is controlled with aerial and ground bait pesticide sprays, but their efficiency depends on knowing the time and location of insect infestations as early as possible in order to initiate the spraying procedure. Producers set up traps in the field that lure and capture Olive flies to detect and manually count them. The producer has to inspect traps throughout most of half the year to identify and selectively count only olive flies. The pest managers are instructed to disregard insects that have been trapped but are not olive flies. Some of them however (e.g., fruit flies) can look very much alike to the untrained eye. Manual counting is tedious as the pest manager must cover long distances since traps are dispersed in not always easily reachable areas. Manual counting is also rudimentary as occasionally hundreds of insects can be found in traps and the managers/farmers practically make only ‘educated guesses’ (see Figure 1). Because of these limitations and the resulting cost not many traps are being deployed, thus limiting the spatial and temporal resolution of collected census data in large areas.

The monitoring task is based on manual counting of trapped pests and depending on the found concentration the spraying procedure is initiated at large spatial scales. The timing of the spraying is critical as initiation prior to and after the optimal time-point returns suboptimal results. The fear of a serious infestation may lead inevitably the producers to compensate the faulty monitoring procedures with excessive spraying of pesticides. Unfortunately, pesticides affect natural enemies of the pests, as well as useful pollinating insects, contaminates water, and carry the risk of exposure to humans. An automated trapping system that would identify, count and transmit measurement from the field to a smartphone would increase census data accuracy, reduce labour expenses associated with manual monitoring, improve capabilities for monitoring larger areas and potentially reduce the amount of spraying.

Monitoring traps are plastic or glass boxes. Insects are attracted to enter the trap by either a pheromone dispenser that hangs inside at the top of the trap or by food baits and the light entering through the clear top. The most famous trap and probably the most widely used is the so called ‘McPhail trap’ consisting of two interlocking sections: (a) a clear top and (b) an inverted funnel. Insects enter the trap through a hole in the inverted funnel base in response to the chemical signals they receive [4]. Generally the reverse movement of the insect out of the trap though not infeasible is uncommon due to the movement patterns of insects and the inverted funnel design. The traps may contain insecticide on their bottom to terminate the pest but typically pests are drowned in the food-bait. We will embed our electronics in this widely known trap as a proof of concept but we do not imply that our approach is in anyway restrained to this trap configuration.

The key component in the electronic McPhail is the opto-electronic sensor. In our approach the sensor is a receptor array of five phototransistors in parallel connectivity and an infrared emitting LED. The use of an optoelectronic device mainly as an experimental instrument to monitor insect movements has been also reported in the past. The first encounter to our knowledge can be traced back to 1955. Richards [5] observed that partial occlusion of the wings of insects flying in front of a solar disk produce fluctuations in the receptor that when analyzed reveal the wing-beat frequency and overtones of the insect and its harmonics. This idea was furthered developed in numerous publications using an optoelectronic device as a means to capture insect movement [6‒9]. The use of microphones was also suggested [10]. There are clear advantages of the use of optoelectronics instead of microphones: (a) the optoelectronic device records an event only when the path from the emitter to the receiver is interrupted whereas a microphone picks up sound from all-directions; (b) optoelectronic devices return a very high signal to noise ratio (are practically noiseless) whereas microphones record all sound sources (e.g., birds, cicadas, wind) and therefore the recordings can become very noisy; (c) microphones although can be protected in several ways against weather conditions, are more vulnerable to open field conditions and finally cost much more. To our point of view, traps carrying microphones are very useful but only for in-lab research.

Though optoelectronic devices have been proposed for several insect applications [11], and their future embedding into traps was foreseen, a review of this literature leaves the reader with little information that could be easily integrated into a product. This could be due to immature technology at that time or the employing of computationally intensive classification techniques that cannot be routinely embedded into small, power-efficient devices [12,13]. The novelty of our paper lies in the following:

(a) A new sensor is implemented using state of the art electronics and details of its hardware components and implementation are given, thus allowing replication. We show that our proposal is cost-effective reaching a total cost below 5 Euros (6.26 $, d.l.v 15/112014).
(b) The sensor as well as the microcontroller responsible for detecting flying insects and transmitting the counts are actually embedded in a widely used trap, thus solving many practical difficulties as encountered in devices operating in real-field, and its function as an integrated whole is studied on the task of recording in-flight the olive-fruit fly wing-beat as well as other insects of interest.

## 2. Experimental Section

### 2.1. The Optoelectronic Sensor

**Figure 2.**
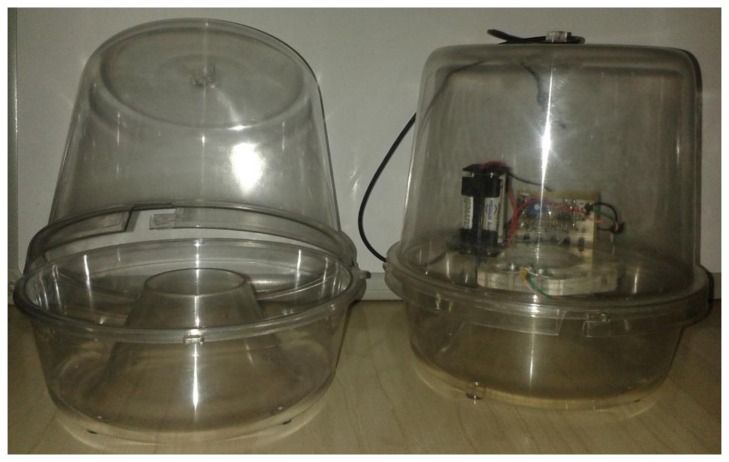
A typical plastic McPhail trap (**Left**) with the optoelectronic sensor embedded (**Right**).

Although many configurations of the McPhail trap are reported in literature, the main components of the trap are the same. In Figure 2 (left) we present a typical plastic McPhail trap with the clear top and the inverted funnel detached. In Figure 2 (right) we show the same trap with all electronics embedded. Herein we give implementation details on the optoelectronic sensor. In our configuration all components are firmly placed on a plexiglas disk cut to fit around the hole of the inverted funnel. The plexiglas base is thick enough to sustain deformations due to temperature variation in real-field conditions. All components are placed on the disk so that their relative position does not change in a possible displacement of the trap due to wind when operating in the real field. Relative position stability of emitter and receiver is a crucial point of the electronic trap.

**Figure 3.**
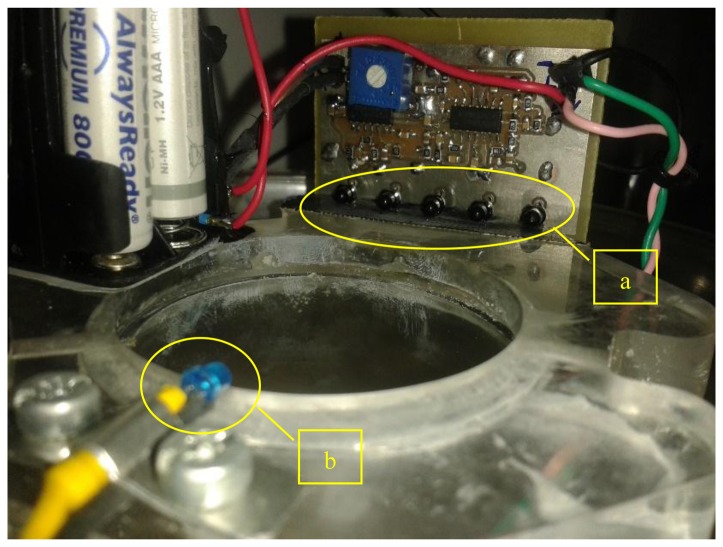
Optoelectronic sensor embedded in the trap. (**a**) the array of phototransistors. A black tape is inserted underneath the phototransistors to cut reflections (**b**) the LED infrared source (blue bulb).

Figure 3 shows an expanded view of the optoelectronic sensor. It is composed of: (a) the emitter which is an infrared light emitting diode (LED); (b) an array of five phototransistors acting as photoreceptors connected in parallel on the other side of the disk. The infrared LED and the phototransistors are in the same package with part number TCRT5000.

**Figure 4.**
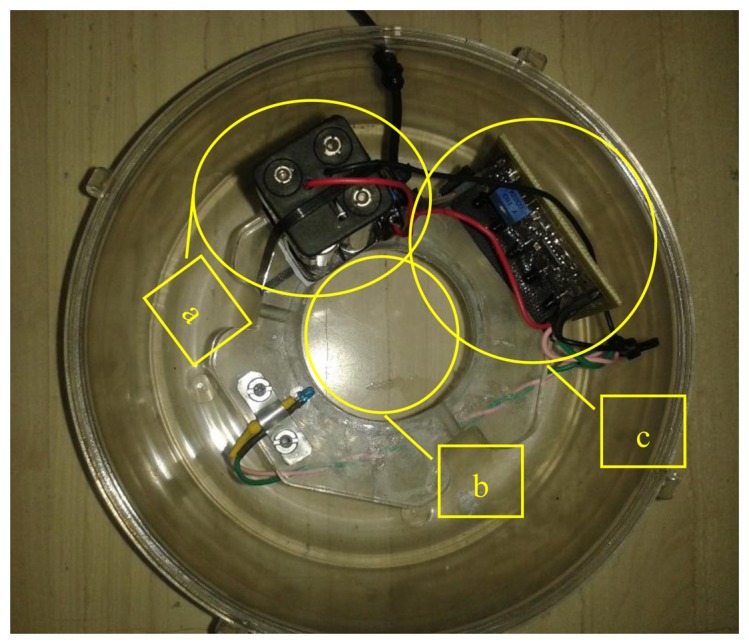
Top view of electronics' placement. (**a**) A 4.8 Volt battery supply on the left; (**b**) the hole in the middle is the entrance of the inverted funnel; (**c**) the array and the LED in their final positioning. All parts sit tight on a Plexiglas disk mounted on the lower part of the McPhail trap.

The remaining components are: An electronic circuit performing band pass filtering (60–4000 Hz), and a 4.8 V power supply from rechargeable batteries and its accompanying switch. A top view of the electronics placement can be seen in Figure 4.

### 2.2. The Electronics

**Figure 5.**
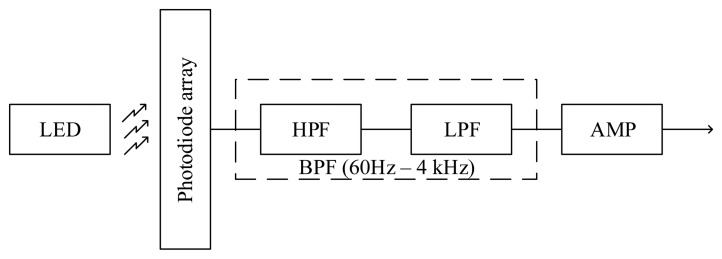
Block diagram of the optoelectronic system.

**Figure 6.**
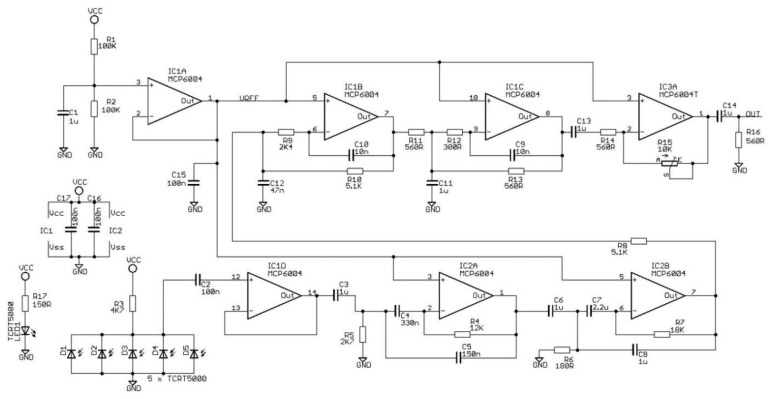
Schematic Description of the filters and operational amplifier attached to the phototransistors. HPF, LPF stands for high-pass, low pass filters respectively and AMP for amplifier.

The optoelectronic system block diagram is shown in Figure 5. It consists of an emitter, a receptor array, a band-pass filter based on a low-pass and a high-pass filter and an adjustable gain amplifier. The electronics board utilizes seven low voltage operational amplifiers (see Figure 6) for signal buffering and the band-pass filter implementation. Specifically, the IC1A is a buffer of half of system's power supply (Vcc/2), creating the reference voltage in order to operate the other amplifiers with a single supply. The IC1D is a buffer for the photoreceptor array signal. The IC2A and IC2B function as a high pass filter with a cutoff frequency of 60 Hz. The IC1B and IC1C function as a low pass filter having a cutoff frequency of 4 kHz. The IC3A is a variable gain amplifier with a maximum gain of 25 dB.

**Figure 7.**
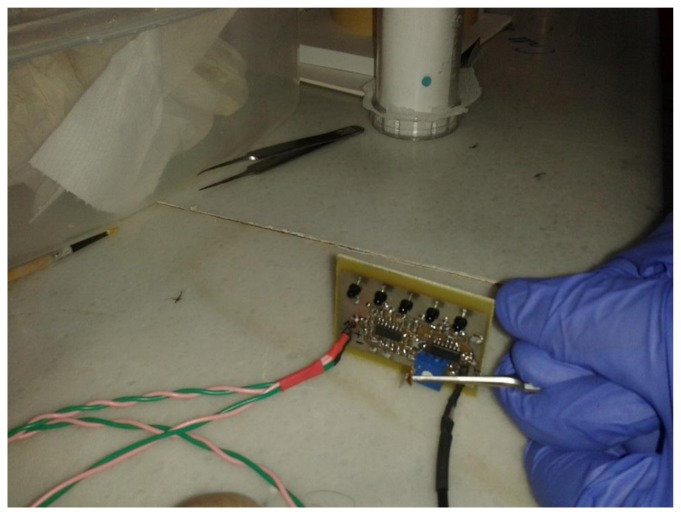
Wing-beat of an olive fly recorded with the optoelectronic sensor.

The signal output is a clear zero-mean recording in the absence of photo path interruption and can easily follow the frequency of wing-flapping in case of insect's presence. The output can be fine-tuned through variable gain amplifier using the potentiometer (R15 in Figure 6, blue square in Figure 3) to be in the range −1 and 1 in order to be treated as line-level audio signal (see Figure 7 for the recording process and Section: *Results and Discussion* for the spectrogram of the recorded signal). The device can operate in daylight as well as in total darkness and the battery supply ensures a steady input that is required for such delicate measurements. This prototype should be operated away from artificial light as the photodiodes are prone to picking up interference.

We are currently looking into a series of design and optimization strategies of photonic devices for sensing applications [14] in order to optimize our sensor in terms of SNR and power consumption.

### 2.3. Capturing of Live Adult Specimens for Controlled and In-Flight Experiments

**Figure 8.**
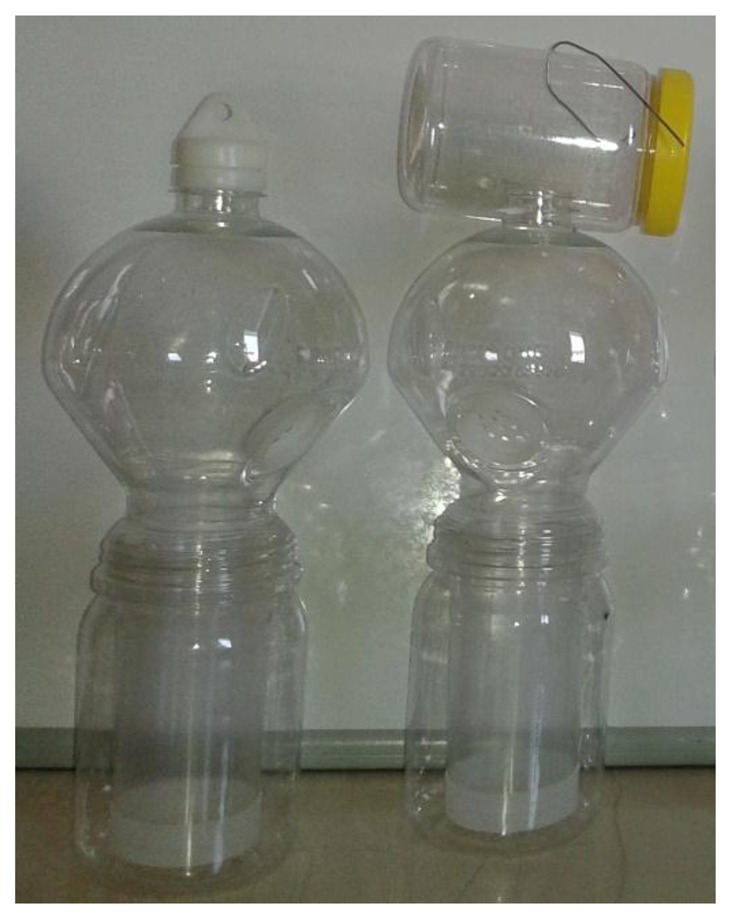
Traps used to capture alive *B. oleae* adults from olive trees. A typical olive-fly trap (**Left**); A custom-made trap with a second room attached (**Right**).

In this paragraph we focus on the sensor but the electronic McPhail will finally include a recognizer built to its electronics that will classify insects from their wing-beat as they fly in the trap. It is well-known in pattern recognition that, in order to achieve optimal classification results, the training and test conditions must be matched. Therefore, we did not use insects that are grown in captivity as we noticed a great difference in their behavioral patterns. Insects grown in captivity are generally reluctant to fly and not as vivid as the naturally grown counterparts. Moreover we did not know if captivity affects other parameters of flight. Therefore we used custom-made traps in order to capture alive *B. oleae* adults from olive orchards as illustrated in Figure 8. The custom-made traps were filled with either pheromone or food baits and hanged from trees. Once inside the trap, the insects have the tendency to fly upwards and therefore we found large numbers of them in the detachable container (Figure 8, right). Insects entering the container can be captured alive otherwise they would be typically drowned in the bait. Once a number of insects are trapped, the container is manually sealed and detached from the main trap while a replacing container is locked in. Subsequently, the removed container is placed in a common freezer for about 1–2 min. *B. oleae* (as well as *Musca domestica* and several mosquito species we tried) are ectotherms meaning that their body temperature is controlled by their outside environment. By putting them in the freezer we instantaneously decrease their activity levels and this allows us to manipulate them and remove other species possibly collected in the trap. Then the detachable container is placed under the Electronic McPhail Trap powered on. After the insects attain the normal temperature of the environment they fly towards the open inverted funnel of the McPhail trap and, therefore we achieve an in-flight recording.

## 3. Results and Discussion

**Figure 9.**
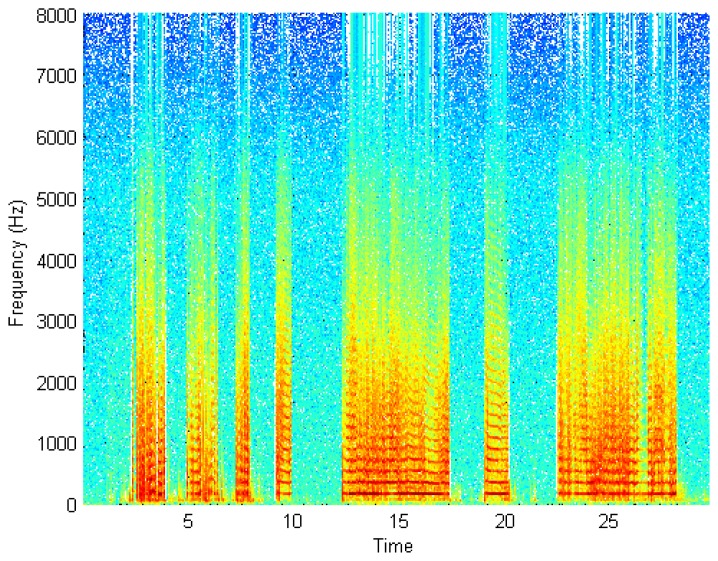
Spectrogram of a recording of *B. oleae* from the optoelectronic sensor.

**Figure 10.**
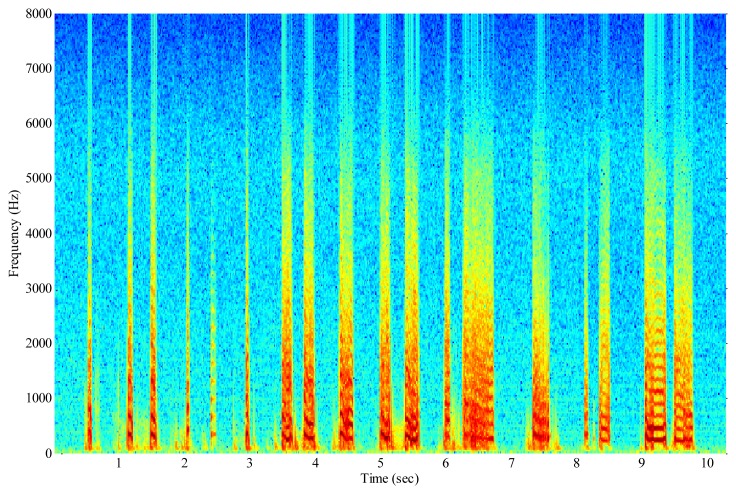
Spectrogram of a recording of common housefly, *Musca domestica* from the optoelectronic sensor. All specimens were manually held in and out of the beam while flapping their wings.

**Figure 11.**
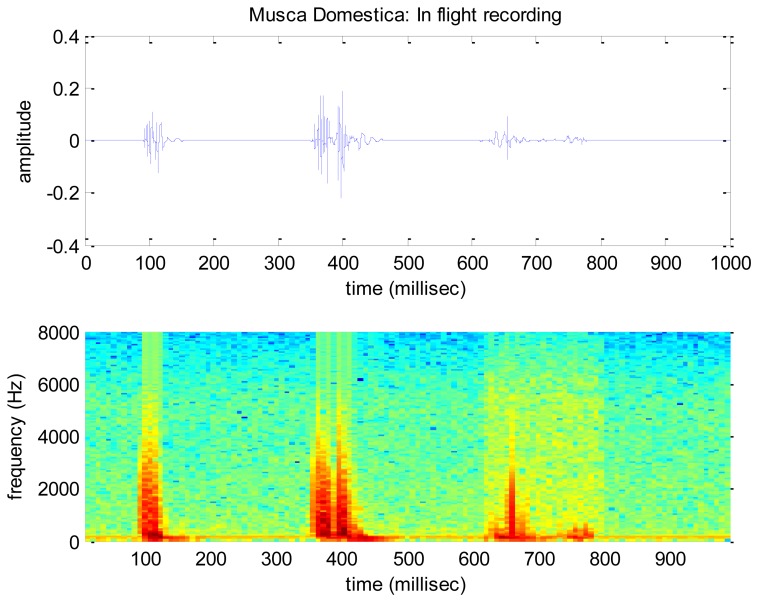
A series of in-flight recordings for *Musca domestica*. The flight time to cross the beam is estimated between 50 and 100 msec.

A spectrogram of a recording of the wing-flap of the insect *B. oleae* is shown in Figure 9. In this figure the recording is sampled at 16 kHz, divided into 512 sample segments with 50% overlap and each segment is windowed with a Hamming window. The specimen is manually held in and out of the infrared beam while flapping its wings. A male adult is shown for the first 10 s and a female adult is shown afterwards. Notice how the fundamental frequency of 180 Hz and the overtones are clearly resolved. Notice also the complete lack of any noise though no signal enhancement method is applied on the recording other than that of the electronic device. A small amount of energy observed in very low frequencies between the 3d and 10th second are actually due to body-movements of the insect. We repeated our controlled experiment with other insects. For economy of space we present results for the common housefly, *Musca domestica*, in Figure 10. The adults are again manually held in and out of the infrared beam while flapping their wings. On the other hand, Figure 11 demonstrates the results of an almost real-field condition experiment we carried out in order to take in-flight recordings by placing a container with pests under the electronic McPhail.

One further experiment we carried out was to compare the recordings of the optoelectronic sensor with that of a common high-quality microphone. Therefore we enclosed in an insectary 20 house-flies and recorded their wing-beat with a typical microphone (Telinga twin-capsule Mono microphone and an Edirol Roland recorder set at sampling frequency of 44.1 kHz). The power spectral density of the recording was derived and compared with the power spectral density of the optoelectronic sensor to cross-check that the readings of the optoelectronic sensor find reasonable accordance with the microphone recordings. Although we took specific care to minimize acoustic interference from other sources we could not avoid that the microphone picked other sounds as well. Moreover, we noticed reverberation due to acoustic reflections in the insectary.

**Figure 12.**
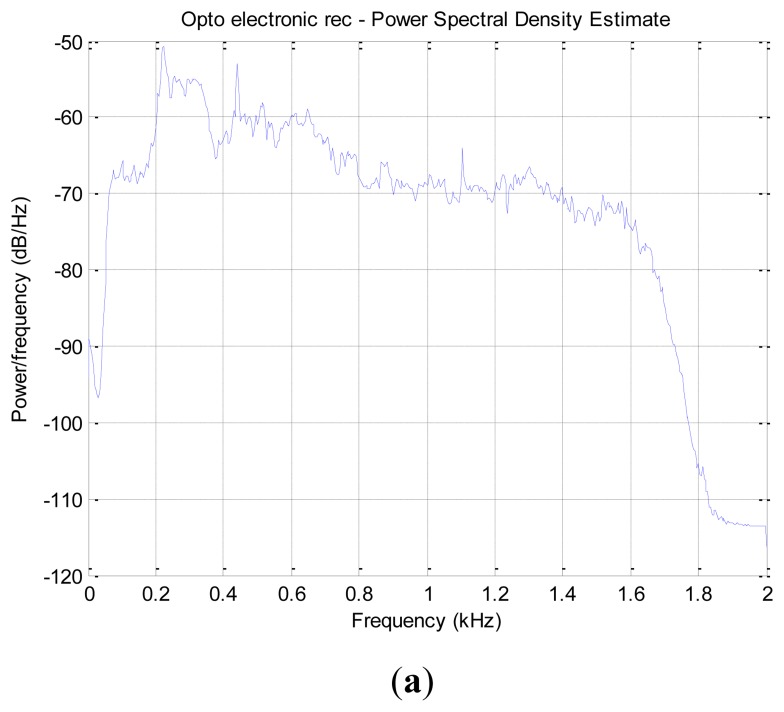

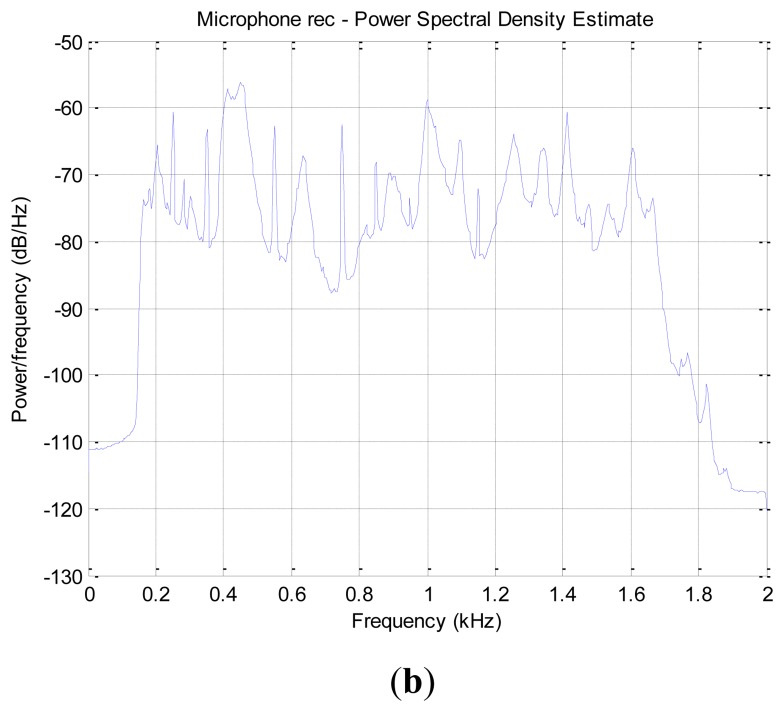
Power spectral densities of recordings of house-flies. (**a**) Recording from optoelectronic sensor; (**b**) Recording taken with a high quality microphone placed inside an insectary hosting 20 house-flies.

This experiment shown as the practical limits of using microphones in the real-field for such delicate applications. The important characteristic that we see in both subfigures of Figure 12 are the frequencies at 220 and 440 Hz (the fundamental and first overtone) being clearly visible in both figures. The microphone seems to be able to capture better a larger number of overtones. Notice however that some energy leakage in certain frequencies are due to acoustic interferences and reverberation. In conclusion, we find that the two recordings are quite similar up to 1 kHz as visually inspection of the spectrogram and hearing of the audio provided both by the optoelectronic device and the microphone.

We support that real-time pest monitoring can be fulfilled because the fundamental frequency and overtones of the wing beat are clearly resolved. Note that most insects beat their wings with frequencies between 100 Hz up to <1 kHz (see [15] for a large collection of wing-beat frequencies). The highest wing beat ever reported for an insect is for the asynchronous muscle system of *Forcipomyia (Diptera: Ceratopogonidae)* [16] attaining a wing beat frequency of 1046 Hz. Wing beats of this order are atypical for most insects that have a beat frequency lower than 300 Hz.

The homo-structured phototransistors (*i.e.*, ones using the same material throughout the device) can typically track frequencies up to 250 kHz but certain hetero-junction devices can reach 1 GHz. Clearly, the operating frequency of phototransistors is far higher than any biological organism can reach with its wings. Therefore, we conclude that optoelectronic devices cannot fail to respond in tracking the wing-beat of any insect.

## 4. Embedding the Event Counter and Species Recognizer

The analog output of the optoelectronic sensor is sent to an Arduino Mega2560 microcontroller platform (Atmel ATmega2560 microcontroller, 16 MHz clock speed, 256 KB Flash, 8 KB SRAM, 4 KB EEPROM) that performs counting of insects passing the beam and recognition of the species. In this work we report results on insect counting.

**Figure 13.**
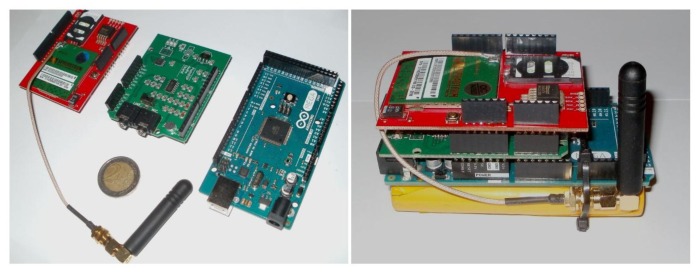
(**Left**) The microcontroller platform and its expansion boards: (from left to right) The GSM expansion board, the audio expansion board and the microcontroller; (**Right**) Assembled microcontroller with battery attached.

**Figure 14.**
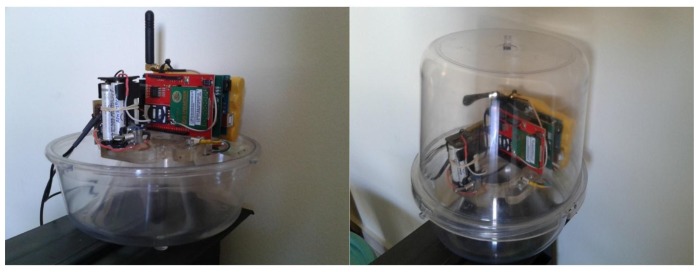
The electronic McPhail trap. (**Left**) Optoelectronic sensor and microcontroller on plexiglass disk; (**Right**) Integrated electronics and trap parts.

The analog signal is captured and, depending on its level, amplified appropriately by an expansion board attached to the microcontroller platform. Capture signals are sampled at 4 kHz, digitized through the boards Analog-to-Digital Converter (ADC) and its root-mean-square values (RMS) are subsequently extracted. The sampling rate is enough to resolve the fundamental frequency of the wing-flap as well several overtones up to 2 kHz. A threshold level on the calculated RMS is set to trigger an event. Because of the high SNR output of the optoelectronic sensor the triggering is very reliable. The platform constantly captures the input from the sensor (storing the data in a circular buffer) but only processes the 512 samples when a triggering event occurs, to avoid overloading the microcontrollers processor and conserve resources (e.g., energy). The 512 samples at 4 kHz sampling rate correspond to a duration of 128 msec that is safely larger than the within-the-beam flight time of 50–100 msec observed from recordings processed offline. The count of trigger events is stored in the device's memory and is transmitted at pre-set intervals (e.g., once a day) via text message to a predefined recipient. The latter is achieved via GSM expansion board which is also attached to the microcontroller. When no signal is present on the input, the board enters sleep mode to conserve resources. The hardware components of the event counter and recognizer can be seen in Figure 13 and the trap with all electronics embedded can be seen in Figure 14.

The cost breakdown for the prototype is depicted in Table 1. It should be noted that, especially in the case of the audio expansion and GSM boards, the total cost is expected to significantly drop when the prototype moves to custom-built hardware (as in their stock form they have many features which are not needed in this application). The trap may look bulky as prototypes usually look but is only constructed this way for the proof of concept. Future plans include customizing the Arduino components and we anticipate that this will significantly reduce the cost and size of the equipment. Moreover we are currently re-designing the classical McPhail trap under different configurations where the electronics are placed as an external add-on the plastic top with minimal disturbance of the internal space.

### Initial Detection Experiments

The detector of events is currently evaluated off-line in the lab. Real field-experiments with insects are expected to take place during the summer and require a careful design and evaluation protocol. We have currently evaluated our system using optoelectronic recordings from the work published in [11]. 100 recordings were constructed from randomly choosing wing-beat events from various insects offered in the associated web-page of [11]. Each recording included one to five events randomly set. These recordings were played back through a media player, the output of which was connected to the microcontroller's audio board input. We did not observe any miss of event in the detected list of events (*i.e.*, the event count logged by the microcontroller) and we attribute this to the high SNR of the optoelectronic sensor readings. We counted an approximate 7.5% of false alarms, as sometimes an entering insect would trigger two events instead of one. We are currently investigating this issue to optimize the performance of the detection algorithm.

## 5. Conclusions

Detection and localization using optoelectronic sensors for agricultural tasks are becoming popular [17] and a valuable component of what is referred as precision agriculture. We constructed a prototype electronic insect trap that can count and record the wing-beat of the olive fly. The optoelectronic sensor is capable of accurate sensing wing-beat characteristics of several insects as well. The following step is to embed a classification algorithm (as in [18]) on the microcontroller platform. We will continue to evolve our sensor prototype, as we did not waterproof it or optimize its power consumption, which is currently 43 mA. The same care will be taken to integrate the various boards of the microcontroller platform and optimize its power consumption. We believe that once optimized the electronic trap has the potential to revolutionize the way insect monitoring is carried out as it returns recordings of very high quality for about 150 € (the sensor costs 5 € and the microcontroller platform's components 150–175 €); a price that is expected to significantly drop in case of wholesale orders and when this proof-of-concept prototype is replaced with custom-made recognizer components (projected price 40 €). Once the insect recordings will be transmitted from the field to the pest manager using the GSM network the cost of maintaining a large number of dispersed traps will be reduced dramatically. The same sensor can be embedded in traps aiming at different insects such as mosquitoes (e.g., alerting for species that are possible carriers of the west Nile virus), bees and fruit flies. In the near future we will report on embedding the classification software on the electronic circuit and transmitting counts and recognition.

## Figures and Tables

**Table 1.** Cost breakdown of the hardware of the electronic McPhail trap. Projected cost refers to custom-made electronics.

**Item**	**Model**	**Current Price € (15/11/2014)**	**Projected Cost (€)**
Sensor	TCRT5000	5	4
Microcontroller	Arduino Mega2560	10–35 (cloned or original)	12
GSM expansion board	SM5100B	80	12
Audio expansion board	PlainDSP	45	4 (custom made)
Batteries	NiMH	10	8

Total		150–175	40

## References

[1] The Olive Oil Sector in the European Union. http://ec.europa.eu/agriculture/publi/fact/oliveoil/2003_en.pdf.

[2] Michelakis S.E. The Olive Fruit Fly (DACUS OLEAE GMEL.) in Crete, Greece. http://www.actahort.org/books/286/286_76.htm.

[3] Economopoulos A.P. (2002). The Olive Fruit Fly, Bactrocera (Dacus) Oleae (Gmelin) (Diptera: Tephritidae): Its Importance and Control; Previous SIT Research and Pilot Testing.

[4] Eliopoulos P.A. (2007). Evaluation of commercial traps of various designs for capturing the olive fruit fly Bactrocera *oleae* (Diptera: Tephritidae). Int. J. Pest Manag..

[5] Richards I. (1955). Photoelectric cell observations of insects in flight. Nature.

[6] Hedwig B. (2000). A highly sensitive opto-electronic system for the measurement of movements. J. Neurosci. Methods.

[7] Engel J.E., Wyttenbach R.A. (2001). An optoelectronic sensor for monitoring small movements in insects. Fla. Entomol..

[8] Gotz K. (1987). Course-control, metabolism and wing interference during ultralong tethered flight in Drosophila Melanogaster. J. Exp. Biol..

[9] Aubrey M., Miller R.H. (2002). Automated identification of optically sensed aphid (Homoptera: Aphidae) wingbeat waveforms. Ann. Entomol. Soc. Am..

[10] Mankin R., Machan R., Jones R. Field Testing of a Prototype Acoustic Device for Detection of Mediterranean Fruit Flies Flying into a Trap.

[11] Chen Y., Why A., Batista G., Mafra-Neto A., Keogh E. (2014). Flying Insect Classification with Inexpensive Sensors. J. Insect Behav..

[12] Moore A., Miller J.R., Tabashnik B.E., Gage S.H. (1986). Automated Identification of Flying Insects by Analysis of Wing-Beat Frequencies. J. Econ. Entomol..

[13] Zhenyu L., Zuji Z., Zuorui S., Qing Y. (2005). Automated Identification of Mosquito (Diptera: Culicidae) Wingbeat Waveform by Artificial Neural Network. Artificial Intelligence Applications and Innovations.

[14] Passaro V.M.N., Tullio C.D., Troia B., Notte M.L., Giannoccaro G., Leonardis F.D. (2012). Recent Advances in Integrated Photonic Sensors. Sensors.

[15] David N.B., Buchmann S., Spangler H. (1988). Relationship between wing loading, wingbeat frequency and body mass in Homopterous insects. J. Exp. Biol..

[16] Scherer C.W. (1995). Chapter 9 Fastest Wing Beat. University of Florida Book of Insect Records.

[17] Garrido M., Perez-Ruiz M., Valero C., Gliever C.J., Hanson B.D., Slaughter D.C. (2014). Active Optical Sensors for Tree Stem Detection and Classification in Nurseries. Sensors.

[18] Potamitis I. (2014). Classifying insects on the fly. Ecol. Inform..

